# Synthetic turf pitches with rubber granulate infill: are there health risks for people playing sports on such pitches?

**DOI:** 10.1038/s41370-018-0106-1

**Published:** 2018-12-19

**Authors:** Marja E. J. Pronk, Marjolijn Woutersen, Joke M. M. Herremans

**Affiliations:** National Institute for Public Health and the Environment (RIVM), Bilthoven, The Netherlands

**Keywords:** Synthetic turf pitches, Rubber granulate infill, Health risks

## Abstract

The presence of carcinogenic substances in rubber granulate made from old car tyres raised concerns that the use of this granulate as infill on synthetic turf pitches may cause leukaemia and lymphoma in young football players and goalkeepers. Limitations in a number of prior studies on the topic casted doubts on their conclusion that it was safe to play sports on such pitches. Rubber granulate samples from 100 Dutch synthetic turf pitches were analysed for 45 (all samples) or 79 substances (a subset). A subset of samples was additionally analysed for migration of polycyclic aromatic hydrocarbons (PAHs), phthalates and metals into sweat and the gastrointestinal tract, and for evaporation of volatile substances into air. Exposure scenarios were developed to estimate the exposure of amateur football players via the oral, dermal and inhalation route to the most hazardous substances in rubber granulate. Risks to human health were assessed by comparing toxicological reference values for these substances with the exposure estimates. A number of carcinogenic, mutagenic and reprotoxic substances were present in rubber granulate used on Dutch pitches. No concern was, however, identified for phthalates, benzothiazoles, bisphenol A and the metals cadmium, cobalt and lead, as their exposures were below the levels associated with adverse effects on health. PAHs appeared to be the substances of highest concern, but even they present no appreciable health risk with exposures resulting in additional cancer risks at or below the negligible risk level of one in a million. Our findings for a representative number of Dutch pitches are consistent with those of prior and contemporary studies observing no elevated health risk from playing sports on synthetic turf pitches with recycled rubber granulate. Based on current evidence, there is no reason to advise people against playing sports on such pitches.

## Background

Synthetic turf pitches (STPs) were initially introduced as alternative for natural grass pitches in the 1960s in the USA and in the 1980s in Europe. The main benefit of STP compared with natural grass pitches is that they can be used intensively, year round, independent of the weather. To give these pitches characteristics and playability comparable to conventional grass, infill material is needed. One type of infill widely used is rubber granulate. Rubber granulate is finely ground rubber, also known as ‘rubber crumb’. Rubber granulate can be made from new synthetic rubber, but also from scrap rubber products, in particular old car tyres. In Europe, a directive banning inert waste such as car tyres from landfills [1] encouraged their recycling as rubber granulate. Granulate made of car tyres is also known as styrene butadiene rubber granulate, or SBR. Although SBR granulate is generally assumed to be made exclusively from car tyres, there is no mandatory verification system, so the end product may include other types of rubber as well. The main use of rubber granulate is as infill material, but it is for instance also used to make shock-absorbing rubber tiles, flooring in playgrounds, and rubber carpeting.

In the Netherlands, the number of STP for football (the European equivalent of soccer in the USA) has gradually grown from a few in the year 2000 to approximately 2000 nowadays. They are exclusively outdoor pitches, and around 90% of them have SBR infill. The remaining 10% have an infill of coated SBR rubber or another infill material, such as ethylene-propylene-diene monomer rubber (EPDM), thermoplastic elastomer (TPE), cork, or mixtures of SBR rubber with various synthetic and natural materials [2]. Besides football pitches there are in the Netherlands also a relatively small number of rugby pitches, korfball pitches and Cruyff courts (small playing fields of synthetic turf in neighbourhoods) with rubber granulate.

Car tyres consist of natural and/or synthetic rubber mixed with a multitude of chemical additives (such as plasticisers, fillers, anti-degradants, vulcanisation substances and reinforcing agents) to give tyres the desired characteristics [3, 4]. Next to substances that are intentionally added during the manufacture of car tyres, there are also impurities in the rubber and the excipients. Additionally, substances may be formed during the production process. Of the many substances present in car tyres and the rubber granulate derived therefrom, some are considered hazardous to people and the environment, e.g., because they are carcinogenic, reprotoxic or accumulate in the food chain. Among these substances are polycyclic aromatic hydrocarbons (PAHs), a number of which are proven carcinogens, as well as a variety of heavy metals, plasticisers and volatile and semi-volatile compounds.

Over the years, the public raised concerns about the safety of recycled tyre rubber crumb used on STP, due to the presence of hazardous substances. Worldwide, several studies have been performed over the last decades to investigate whether there is indeed an elevated health risk from playing on STP with this kind of infill. A review of available literature on these studies concluded that overall, there was no such risk [5]. A number of studies expressed reservations though, acknowledging study limitations such as lack of reliable exposure data, limited number of samples and/or limited number of substances investigated. The absence of (cancer) epidemiology studies and biomonitoring data was also pointed out [6]. Uncertainty around the safety of rubber infill therefore remained, and the topic is still on the research agenda today.

Early 2016, for example, US Environmental Protection Agency (EPA), the Centers for Disease Control and Prevention/Agency for Toxic Substances and Disease Registry (ATSDR) and the Consumer Product Safety Commission (CPSC) launched a multi-agency action plan called the ‘Federal Research Action Plan on Recycled Tire Crumb Used on Playing Fields and Playgroundsʼ [7] to study key environmental and human health questions. This large-scale, comprehensive investigation seeks to ‘fill important data and knowledge gaps, characterise constituents of recycled tyre crumb, and identify ways in which people may be exposed to tyre crumb based on their activities on the fieldsʼ [8, 9] and is expected to substantially contribute to insights into the risks from tyre crumb. Another recent long-term research study on the possible health risks of rubber granulate was initiated by the Californian Office of Environmental Health Hazard Assessment (OEHHA) in 2015 [10]. This study not only includes literature research and chemical characterisation of rubber granulate, but further aims to develop exposure scenarios and to include biomonitoring of sports players as part of the exposure assessment. Finally, in June 2016 the European Chemicals Agency (ECHA) was requested by the European Commission to evaluate whether the presence of certain substances in rubber granulate made from scrap tyres could lead to health risks that are not adequately controlled and require additional measures on EU level [11]. Based on the current evidence available, ECHA concluded early 2017 that there is a very low level of concern from exposure to substances found in the granulate [12].

In the Netherlands, the topic experienced a sudden increase in public interest following a broadcast of a Dutch TV programme in October 2016. A possible link was suggested between carcinogenic substances present in rubber granulate made from car tyres and the occurrence of leukaemia and lymphoma in young football players, goalkeepers in particular. Furthermore, doubts were casted on conclusions from previous investigations that it was safe to play sports on STP with rubber granulate, as it was claimed that overall, there has been too limited research into the health risks. The broadcast resulted in a widespread societal concern, with interested parties (parents, sports clubs, ministries, municipalities, public health services) demanding answers on the safety, on whether or not to close down the pitches, and on what precautionary measures to take. While noting that international large-scale investigations into the health risks of rubber granulate were ongoing, the public concerns expressed were such that the Minister of Health, Welfare and Sport decided not to await their results but requested RIVM to immediately conduct a research on national level. This research was to include sampling of 50–100 Dutch pitches, chemical analyses into the content of the rubber granulate samples, evaluation of the international scientific literature into the hazards of rubber granulate and substances present therein, and assessment of the health risks. The main questions to be answered were: is it safe to play sports on STP with rubber infill, and is there a need for immediate action?

As the time given for the research was only 2 months, targeting was inevitable. The efforts following sampling of 100 Dutch pitches were therefore directed at the chemical risks of rubber infill, not at, e.g., risks from microbiological contamination or heat stress, or risks associated with other types of infill or the synthetic turf itself. The literature was reviewed for substances reported to be often present in tyres and rubber infill, and a number of these were selected for chemical analyses. The risk assessment subsequently focussed on a subset of prioritised substances (i.e., on substances that have hazardous properties considered to be of most concern for humans), and on children and adults playing amateur football. Very small children (below 4 years of age), professional football players and workers installing and maintaining the fields were thus excluded for risk assessment, nor were environmental risks addressed. The latter was subject of additional research [13]. An external scientific advisory group reviewed the targeted approach and the final report to ensure scientific soundness. This article presents the approach and outcome of the investigation. A detailed account of the methodology and results is published in the scientific background document of the RIVM report ‘Evaluation of health risks of playing sports on synthetic turf pitches STPs with rubber granulateʼ [14].

## Methods

### Sampling

In order to gain insight into the substances comprised in rubber infill made from old car tyres, samples were taken of 100 randomly selected STP in the Netherlands (96 football pitches, 2 korfball pitches and 2 Cruyff courts, all outdoor). This sample size represents over 5% of the total number of STP with SBR infill in the Netherlands. With six positions per pitch sampled, a total of 600 rubber granulate samples were collected. Out of the 100 pitches, 10 were sampled in triplicate to collect extra samples for counterchecks (60 samples) and additional investigations (60 samples). After sampling, it appeared that the infill of 9 out of 100 pitches (with 3 out of 9 in the subset of 10 pitches) consisted entirely or partly of material other than car tyre rubber. The samples from these pitches were eliminated from the data set, resulting in a total data set of 546 samples (91 pitches × 6 positions), with a subset of 42 samples (7 pitches × 6 positions) [14].

### Analyses

All 546 rubber granulate samples were analysed for a standard set of 45 substances (16 PAHs, 7 phthalates, 6 volatile organic compounds (VOCs) and 16 metals; Table 1a). Metals were analysed for leaching into water (following shaking a mixture of rubber granulate and water for 24 h at room temperature), all other substances for content. The subset of 42 samples was counterchecked for PAH and phthalate content and metal leaching. This subset (or part thereof) was also analysed for 34 additional substances of interest that for practical reasons could not be included in the standard set (phenols, polychlorinated biphenyls (PCBs), benzothiazoles, and other PAHs and phthalates; Table 1b). Further analyses on some of the subset samples included a ‘general unknown screening’ and three types of migration experiments. For the ‘general unknown screening’ (to detect possible other substances of interest present in rubber granulate that were not actively sought for), rubber granulate extracts were analysed by gas chromatography and mass spectrometry. The peaks were subsequently compared with a library of standard reference materials of the National Institute of Standards and Technology and given a name and CAS registry number.

**Table 1:** Substances analysed for content or leaching into water (metals only)

(a) Standard set			
Metals (leaching)	PAHs	Phthalates	VOCs
Antimony	Acenaphtene	Dihexyl phthalate (DHP)	Benzene
Arsenic	Acenaphtylenene	Dimethyl phthalate (DMP)	Toluene
Barium	Anthracene	Diethyl phthalate (DEP)	Ethylbenzene
Cadmium	Benzo[a]anthracene (BaA)	Di-n-butyl phthalate (DBP)	o-Xylene
Chromium	Benzo[a]pyrene (BaP)	Diisobutyl phthalate (DIBP)	p- and m-Xylene
Cobalt	Benzo[b]fluoranthene (BbFA)	Butyl benzyl phthalate (BBP)	Styrene
Copper	Benzo[k]fluoranthene (BkFA)	Di(2-ethylhexyl) phthalate (DEHP)	
Mercury	Benzo[g,h,i]perylene		
Lead	Chrysene (CHR)		
Molybdenum	Dibenzo[a,h]anthracene (DBahA)		
Nickel	Phenanthrene		
Selenium	Fluoranthene		
Tin	Fluorine		
Titanium	Indeno[1,2,3-cd]pyrene		
Vanadium	Naphthalene		
Zinc	Pyrene		
**(b) Additional set**			
**Metals**	**PAHs**	**Phthalates**	**VOCs**
—	Benzo[c]fluorene	Diphenyl phthalate (DPP)	—
	Benzo[e]pyrene (BeP)	Diisononyl phthalate (DINP)	
	Cyclopenta[c,d]pyrene	Diisodecyl phthalate (DIDP)	
	5-Methylchrysene	Di-n-octyl phthalate (DNOP)	
	Dibenzo[a.l]pyrene	Di-n-nonyl phthalate (DNNP)	
	Dibenzo[a,e]pyrene	Dicyclohexyl phthalate (DCHP)	
	Dibenzo[a,i]pyrene	Bis (2-ethylhexyl) adipate (DEHA)	
	Dibenzo[a,h]pyrene		
**Phenols**	**PCBs**	**Benzothiazoles**	
4-t-Octylphenol	PCB28	Benzothiazole	
4-Nonylphenol	PCB52	2-Hydroxybenzothiazole	
Bisphenol A (BPA)	PCB101	2-Mercaptobenzothiazole (2-MBT)	
Triclosan	PCB118	2-Methoxybenzothiazole	
	PCB138	2-Aminobenzothiazole	
	PCB153	2,2-Dithiobis(benzothiazole)	
	PCB180	N-Cyclohexyl-1,3-benzothiazole-2-amine	
		N-Cyclohexyl-2-benzothiazole sulphenamide	

(a): Substances tested in all 546 rubber granulate samples. (b): Substances tested in subset of 42 (phthalates only) or 7 rubber granulate samples

*PAHs* polycyclic aromatic hydrocarbons, *PCBs* polychlorinated biphenyls, *VOCs* volatile organic compounds

The migration experiments focused on the migration of PAHs, phthalates and metals from rubber granulate into (artificial) sweat and gastrointestinal juices, and on volatile substances that could evaporate from rubber granulate under warm weather conditions. For the migration into sweat, rubber granulate was covered with artificial sweat and left to stand in a Petri dish for 2 h at 37 °C. The Tiny-TIM model [15] was used to simulate the digestion of rubber granulate in the gastrointestinal tract. This model is an in vitro system consisting of two compartments that simulate the conditions in the stomach and the small intestine. During the experiment, peristalsis is simulated in both compartments for a total of 4 h at 37 °C, upon addition of artificial saliva, gastric and intestinal juices. With headspace analysis, the evaporation of volatile substances was determined following heating of rubber granulate for minimally 6 h at 60 °C, using a standard mixture of 65 VOCs as reference [14].

The experimental protocol did not include measurements for PM10 (particulate matter of particles smaller than 10 μm in diameter) or migration of substances out of rubber granulate dust into artificial lung fluid. To simulate dust exposure, literature data on PM10, as measured at indoor STP with SBR infill, were used [16].

### Prioritisation of substances and hazard assessment

Prioritisation of substances present in rubber infill made from old car tyres was done in a two-step approach. In the first step, the content data for all substances analysed were compared with European (or Dutch) regulatory limit values of possible relevance for (substances in) rubber granulate. There is no legislation in Europe or the Netherlands that specifically applies to rubber granulate. However, in the EU rubber granulate is considered a mixture and as such it has to comply with the rules under the REACH (Registration, Evaluation, Authorisation and Restriction of Chemicals) and CLP (Classification, Labelling and Packaging) Regulations [17, 18]. Of particular relevance are REACH Annex XVII entries 28–30, specifying the maximum concentration limits for carcinogenic, mutagenic or reprotoxic (CMR) substances in mixtures. Had rubber granulate been considered an article rather than a mixture, as for instance is the case for shock-absorbing rubber tiles, then other REACH Annex XVII entries (e.g., entry 50 on eight specific PAHs, called ECHA-8 further on) would apply to certain substances present in rubber granulate. Hence, also the limit values specified therein, although not directly applicable to rubber granulate, have been included in the comparison. Further, limit values specified in the Toy Safety Directive [19] and the Soil Quality Decree [20] for some substances present in rubber granulate have been included, given the comparable material or field of application. The philosophy behind including limit values from legislation not formally applicable to rubber granulate is that when the concentration of a substance in rubber granulate does not exceed the limit value(s) considered acceptable for that particular substance in products/media other than rubber granulate, this concentration should also be acceptable for rubber granulate. Therefore, only those substances from Table 1a, b for which the maximum pitch concentration found exceeded one or more regulatory limit values were considered priority substances in the first step.

The substances prioritised in step 1, as well as the substances for which no regulatory limit value was available, were further prioritised in step 2. The criterion used in this step was the classification of the substance as CMR category 1A (for a proven CMR in humans) or 1B (for a probable CMR in humans) in the Classification & Labelling Inventory [21]. This inventory is a public database containing the classifications (whether officially laid down in European legislation or self-applied by companies) for all notified and registered chemical substances in Europe. The prioritisation was for substances with a CMR profile as generally these hazards are considered to constitute the properties of highest concern for humans.

Time constraints did not allow a complete hazard assessment of the prioritised substances, including the setting of toxicological reference values to be used for risk assessment. Therefore, literature was searched for toxicological reference values derived or used by international scientific committees or organisations in their risk assessment of these substances.

### Exposure scenarios and exposure assessment

For children and adults playing amateur football, exposure scenarios were developed to estimate their potential exposure to substances in rubber granulate via the oral (through accidental ingestion), dermal (through skin contact) and inhalation route (through inhalation of vapours or rubber dust). A distinction was made by age, by intensity of playing (recreational or performance-oriented), by position (field player or goalkeeper) and by total duration of playing football (during a specific age category, or ‘lifelong’). This resulted in a total of five scenarios:

1. Field player aged 4–11 years (recreational).
2. Goalkeeper (from 7 years of age).
3. Field player aged 11–18 years (performance oriented).
4. Field player aged 18–35 years (performance oriented).
5. ‘Lifelong’ field player (scenarios 1 + 3 + 4, plus additional ‘veteran’ scenario, i.e., recreational field player aged 36–50 years) or ‘lifelong’ goalkeeper (scenario 1 (age 4–6) plus scenario 2 (age 7–50)).

For the exposure estimation, simple, first-tier equations were used. These equations, including the values used for the input parameters, are presented in Table 2. Migration data (or, when absent for a substance, content data) were taken from the analytical analyses performed. Values for the frequency and duration of playing football were determined in consultation with the Royal Dutch Football Association [2]. Values for the other input parameters were derived from literature. In the absence of literature data on the amount of rubber grains ingested or in contact with the (intact or damaged) skin during playing football, these values are largely based on reported data on contact with soil. The input parameters were chosen in such a way that a worst-case exposure estimate is derived, thereby assuming that each and every training session and match will take place on an STP with rubber granulate, from which each time the maximum amount of substances present will be released.

**Table 2:** Equations and input parameters for exposure calculations

	Scenario 1—Field player 4–11 yr	Scenario 2—Goalkeeper from 7 yr	Scenario 3—Field player 11–18 yr	Scenario 4—Field player 18–35 yr	Reference
**General**					
Body weight (kg)^a^	15.7	24.3	44.8	68.8	[51]
Frequency (times per week)	2	3	5	5 (veterans: 2)	[2]
Duration (hours per event)	1 × 1 h, 1 × 1.5 h	1 × 1 h, 2 × 1.5 h	1.5	2	[2]
Duration^b^ (months per year)	7 (all routes)	7 (dermal) 10 (inhalation and oral)	7 (dermal) 10 (inhalation and oral)	7 (dermal) 10 (inhalation and oral)	[2]
**Dermal** Daily exposure = [mass of granulate in dermal contact] × [migrated content]^c^/[body weight] Year average exposure = daily exposure × [frequency (times) per week] × [frequency (months) per year]					
Uncovered body surface area potentially in contact (cm^2^)^a^	1260 (¼ legs, ½ arms, hands)	1290 (¼ legs, ½ arms)	2680 (¼ legs, ½ arms, hands)	3680 (¼ legs, ½ arms, hands)	[51]
Amount of rubber granulate on skin (g)^d^	1	10^e^	3.3	6	[29]
**Inhalation** (rubber dust)^f^ Daily exposure to rubber dust = [PM10] × [content] × [respiratory rate] × [duration (hours) per event] Year average exposure = [PM10] × [content] × [respiratory rate] × [duration (hours) per week] × [frequency (months) per year]					
Respiratory rate (m^3^/h; for intensive activity)	1.58	1.92	2.53	3.07	[51]
PM10 (µg/m^3^) rubber granulate^g^	12	12	12	12	[16]
**Oral** Daily exposure = [mass of ingested granulate] × [migration data for substance]^c^/[body weight] Year average exposure = daily exposure × [frequency (times) per week] × [frequency (months) per year]					
Amount of rubber granulate ingested (g)	0.2	0.2	0.05	0.05	[52]
**Scenario 5** ‘Lifelong’ field player exposure = [year average exposure scenario 1 × 7 years/70 years] + [year average exposure scenario 3 × 7 years/70 years] + [year average exposure scenario 4 × 18 years/70 years] + [year average exposure veteran scenario × 16 years/70 years]					
‘Lifelong’ goalkeeper exposure = [year average exposure scenario 1 × 3 years/70 years] + [year average exposure scenario 2 (bw 24.3 kg) × 4 years/70 years] + [year average exposure scenario 2 (bw 44.8 kg) × 7 years/70 years] + [year average exposure scenario 2 (bw 68.8 kg) × 18 years/70 years] + [year average exposure scenario 2 (bw 68.8 kg, veteran) × 16 years/70 years]					

*PM10* particulate matter of particles smaller than 10 μm in diameter

^a^For scenarios 1, 2 and 3, the data represent values for the youngest age within the age group. Per kg of body weight, this results in a worst-case exposure estimate for the age group

^b^Excluding a 2-month summer break, and additionally for scenario 1 a 3-month winter break. For the other scenarios, skin of legs, arms and hands will be covered during winter season, resulting in a total of 7 months dermal exposure rather than 10 months

^c^Or content value, when no migration data are available for a substance

^d^One gram of rubber granulate amounts to approximately 12 cm^2^, when spread out in a 1-grain thick layer, so per cm^2^ the skin can be exposed to 0.083 g rubber granulate. The literature data on amount of rubber granulate in contact with skin represent approximately 1, 1.4 and 2% of the total area that can potentially be in contact in scenarios 1, 3 and 4, respectively

^e^In the absence of literature data for goalkeepers, a 10 times higher exposure than for scenario 1 was assumed, corresponding to approximately 10% of the total area that can potentially be in contact with skin

^f^Inhalation exposure via vapours was not part of the exposure estimation as none of the prioritised substances was included in the headspace analysis

^g^Indoor value

### Risk assessment

For risk assessment, the exposure estimates calculated for the various scenarios and prioritised substances were compared with the toxicological reference values for these substances. The resulting risks per exposure route were subsequently summed in order to estimate the combined risk over all routes.

For prioritised substances for which the toxicological reference value is based on a non-threshold effect (such as for the PAHs), the comparison solely concerned the ‘lifelong’ exposure estimate. For prioritised substances for which the toxicological reference value is based on a threshold effect, a tiered approach was followed: only if the risk assessment based on the daily exposure estimates resulted in a cause for concern (i.e., the risk characterisation ratio (RCR) between the daily exposure and the toxicological reference value was greater than one), then further comparisons on the basis of year average or ‘lifelong’ exposure estimates were done. For substances for which the daily exposure estimates did not result in an RCR > 1 this was not necessary, as the daily exposure estimate is more worst case than the year average estimate and the ‘lifelong’ exposure estimate.

## Results

### Substances in rubber granulate sampled from Dutch STP

The chemical analyses of rubber granulate from Dutch STP show various PAHs, phthalates, benzothiazoles and phenols in a large number of the rubber granulate samples. Some of the samples also have low concentrations of PCBs. Volatile substances, however, were almost not found (benzene, ethylbenzene and o-xylene in none of the samples, toluene, p- and m-xylene and styrene only in one or two samples, at levels just above the limit of detection of 0.05 mg/kg). No content analyses were done for metals, but from literature and the leaching experiment it is clear that various metals are present in rubber granulate, zinc in particular. Overall, there was little variation in the concentrations of analysed substances between the pitches and between the six positions per pitch (data not shown). Supplementary Table S1 presents an overview of the substances that are detected in at least 5% of the samples analysed for these substances. In this table also, the comparison with the regulatory limit values is presented.

The comparison shows that the rubber granulate on all tested pitches complies with the limits applicable for mixtures: for all CMR substances in rubber granulate, the concentrations found (either for the individual substance or for the group) were all well below the concentration limits applicable for mixtures. For the carcinogenic PAHs for instance, the concentrations found for ECHA-8 (5.8 and 19.8 mg/kg for the 50th percentile and maximum of the pitch values, respectively) were considerably lower than the concentration limits of 100 or 1000 mg/kg specified in REACH Annex XVII entry 28 for individual members of this group. However, other regulatory limit values, although not directly applicable to rubber granulate as such, were exceeded by some substances in rubber granulate. This concerned five of the eight PAHs that are subject to limits in consumer articles and toys under REACH Annex XVII entry 50: BaP, BaA, CHR, BbFA and BeP exceeded both limits (1 and 0.5 mg/kg, respectively) by 2.2–7.75 and 4.4–15.5-fold, respectively. Two phthalates (DEHP and DIBP) exceeded the soil limit, but none of the six phthalates that are subject to REACH Annex XVII entry 51/52 exceeded the limit value for toys. Finally, three metals (cadmium, cobalt and lead) exceeded the limit value specified by the Toy Safety Directive (with cadmium and cobalt also exceeding the soil limit). However, the limit in the Toy Safety Directive concerns a migration limit and the comparison with the content is a worst-case scenario, since it is unlikely that 100% will migrate. Indeed, the migration into water appeared limited to absent for these metals (Supplementary Table S2).

In order to detect possible other substances of interest in rubber granulate that were not part of the analyses, the experimental protocol included a ‘general unknown screening’ on samples from three different pitches. Due to lack of time in the 2-month research period in 2016, this screening was performed in 2017. Comparison of the mass spectra with a library of standard reference materials of the National Institute of Standards and Technology resulted in a list with CAS registry numbers for 183 ‘unknown’ peaks. For all 183 substances, it was checked whether they fulfilled the second prioritisation criterion (classification as CMR category 1A/1B), to see whether additional confirmatory identification and quantitative analysis was necessary. Only for one of the 183 substances such an analysis was needed. This substance was 2-methyl-2-butene (cas nr. 513-35-9), which has no official classification as CMR 1A/1B but is self-classified by companies as carcinogen 1B. The result of the confirmatory identification analysis was negative, leading to the overall conclusion that no additional substances of interest were present in rubber granulate [22].

### Prioritised substances for risk assessment

Substances that were prioritised following the two-step approach are presented in Table 3, together with the toxicological reference values used for risk assessment. Some additional PAHs and phthalates (marked in italics in Table 3) were also included for risk assessment, the PAHs because they were included in the toxicological reference value, the phthalates because most phthalates are reprotoxic and have the same mode of action, so combined toxicity is likely.

**Table 3:** Substances prioritised for risk assessment and toxicological reference values identified

Substances	Abbreviation	Cas no.	Toxicological reference value			Ref.
			Oral (mg/kg bw/d)	Dermal (mg/kg bw/d)	Inhalation (mg/m^3^)	
Metals						
Cadmium		7440-43-9	2.5 × 10^−3^ mg/kg bw/wk (TWI)		5 × 10^−6^ (AQGV)	[53, 54]
Cobalt		7440-48-4	1.4 × 10^−3^ (TDI)		0.5 × 10^−3^ (TCA)	[55]
Lead		7439-92-1	0.05 × 10^−3 a^		0.5 × 10^−3^ (AQGV)	[56–58]
PAHs						
Benzo[a]pyrene	BaP	50-32-8			1 × 10^−6 b^	[59]
Benzo[a]anthracene	BaA	56-55-3				
Chrysene	CHR	218-01-9				
Benzo[b]fluoranthene	BbFA	205-99-2				
* Benzo[k]fluoranthene*	BkFA	207-08-9				
* Dibenzo[a,h]anthracene*	DBahA	53-70-3				
Benzo[e]pyrene	BeP	192-97-2				
* Benzo[j]fluoranthene*	BjFA	205-82-3				
	= ECHA-8		0.49^c^ (BMDL_10_)	0.74^c^ (BMDL_10_)		[14, 38]
Phthalates						
Bis(2-ethylhexyl) phthalate	DEHP	117-81-7	0.034 (DNEL)	0.672 (DNEL)	0.12 (child) 0.16 (adult) (DNELs)	[60, 61]
Diisobutyl phthalate	DIBP	84-69-5	0.0083 (DNEL)	0.08 (DNEL)	0.025 (DNEL)	[60, 62]
* Dibutyl phthalate*	DBP	84-74-2	0.0067 (DNEL)	0.07 (DNEL)	0.02 (DNEL)	[60, 63]
* Benzyl butyl phthalate*	BBP	85-68-7	0.5 (DNEL)	10 (DNEL)	1.7 (DNEL)	[60, 64]
* Diisononyl phthalate*	DINP	28553-12-0	0.25 (DNEL)	6.25 (DNEL)	0.87 (child) 1.16 (adult) (DNELs)	[65]
Dicyclohexyl phthalate	DCHP	84-61-7	0.18 (DNEL)	1.8 (DNEL)	0.63 (DNEL)	[14]
Other						
2-Mercaptobenzothiazole	2-MBT	149-30-4	0.31 (DNEL)	0.94 (DNEL)	1.09 (DNEL)	[66]
Bisphenol A	BPA	80-05-7	4 × 10^−3^ (DNEL)	0.1 × 10^−3 d^ (DNEL)	0.2 (DNEL)	[14, 67]

Substances additionally selected subsequent to the two-step prioritisation approach are given in italics

*AQGV*  air quality guideline value, *BMDL*_*10*_  95% lower confidence level of the dosage resulting in a 10% additional cancer risk in laboratory animals upon lifelong exposure, *DNEL*  derived no-effect level, *TCA* tolerable concentration in air, *TDI* tolerable daily intake, *TWI * tolerable weekly intake

^a^Related to developmental neurotoxicity, a non-threshold effect. The reference value is a maximum exposure value

^b^Related to non-threshold carcinogenicity. The reference value is for BaP, as marker for a PAH mixture, and relates to an additional cancer risk of 1 × 10^−4^

^c^Related to non-threshold carcinogenicity of a PAH mixture. The reference value is for the sum of the eight PAHs specified (the so-called ECHA-8, i.e., the eight PAHs in REACH Annex XVII, entry 50). The reference values relate to additional cancer risks of 1.43 × 10^−3^ and 9.46 × 10^−4^ per µg/kg bw/d for the oral and dermal route, respectively

^d^Internal value (adjusted for absorption)

All toxicological reference values, with the exception of those for PAHs and lead, are related to a threshold effect. For PAHs, they are related to genotoxic carcinogenicity, a non-threshold effect. The Dutch policy on genotoxic carcinogenic substances is to aim at achieving a negligible or maximum permissible risk level on lifelong exposure. The negligible risk level is one in a million (1 × 10^−6^, i.e., one additional case of cancer per million exposed individuals), whereas the maximum permissible risk level is one in 10 thousand (1 × 10^−4^, one additional case of cancer per 10 thousand exposed individuals). For inhalation, the toxicological reference value of 1 ng/m^3^ relates to an additional cancer risk of 1 × 10^−4^, with BaP as marker for the total PAH mixture in air. The oral and dermal toxicological reference values for the PAHs are for the sum total of the eight PAHs included in REACH Annex XVII entry 50 (ECHA-8) as marker for the total PAH mixture in rubber granulate. For risk assessment, a grouping approach was therefore followed, summing the estimated exposures over the ECHA-8 PAHs per route of exposure. In accordance with the REACH guidance [23], linear extrapolation was subsequently used to express the summed exposures in terms of additional cancer risk. This resulted in additional cancer risks of 1.43 × 10^−3^ and 9.46 × 10^−4^ per µg/kg bw/d for the oral and dermal route, respectively [14].

The toxicological reference value for lead also relates to a non-threshold effect. It is based on developmental neurotoxicity, and the reference value was set extremely low to minimise lead exposure to a level as low as possible.

### Results of migration tests

The headspace analysis was performed to measure the release of VOCs from rubber granulate under warm weather conditions. VOCs like benzene, toluene, ethylbenzene, xylene, styrene and 1,3-butadiene were not found in levels above the limit of detection in the evaporated air. Other VOCs, such as ethanol, acetone, carbon disulphide, acetaldehyde, methyl ethyl ketone and methyl isobutyl ketone, were found to a limited extent. Using a dispersion model and worst-case assumptions (a combination of high solar radiation, high temperature and low wind speed), the evaporated substances were calculated to be present in only relatively low concentrations in the air above an STP [14]. Although only a limited number of samples were tested, the results seem to indicate that inhalation of vapour with VOCs from rubber granulate does not contribute significantly to the exposure of people playing football. No headspace analyses were available for the prioritised substances. To have some indication for PAHs, a model calculation at 60 °C for BaP (using vapour pressure and maximum pitch concentration of BaP as input) revealed a maximum concentration of 0.03 ng BAP/m^3^ in the air immediately above the STP. This concentration is considered worst case (in practice, very high air temperatures do not occur very often in the Netherlands, and the wind would disperse some of the substance), and is well below the European limit value of 1 ng/m^3^ for BaP (Table 3).

Supplementary Table S2 shows the results of the leaching into water of the metals, as well as the results of the in vitro digestion and skin migration tests performed for some PAHs, phthalates and metals. From a large number of rubber granulate samples, zinc, copper, cobalt and barium leached into water. The extent of migration was, however, 150–560-fold lower for copper, cobalt and barium than for zinc. Whereas for zinc this could possibly result in environmental effects, zinc is not of concern to human health (not CMR) and therefore not selected for risk assessment. Since for most metals, including the prioritised ones (cadmium, cobalt and lead), leaching to water was limited to absent, skin exposure to metals in rubber granulate via rainwater will in all likelihood not be an important exposure route for people playing football during/after rainfall.

Simulated digestion of the rubber granulate samples in the Tiny-TIM model showed the migration of several PAHs, phthalates and metals into artificial gastric/intestinal juices. PAHs and metals, but not phthalates, also migrated into artificial sweat. When comparing the content data for the detectable PAHs and phthalates to their migration levels, it was estimated that after ingestion approximately 20% of the phthalates and 9% of the PAHs is released into the gastrointestinal tract, whereas only approximately 0.02% of the PAHs is released into sweat. For metals, this comparison was not possible given the lack of content data. Since the results of the migration tests relate only to a limited number of samples, the concentrations found do not provide a comprehensive overview of the range of concentrations that can possibly be found for all Dutch pitches sampled. However, in the samples tested there appeared to be a fairly robust relationship between the content and migration level, for both PAHs and phthalates. This led to a tentative assumption that 9% of the PAHs and 20% of the phthalates migrate from the rubber granulate into the gastrointestinal tract, and 0.02% of the PAHs into sweat. These percentages were therefore used to estimate the migration for all PAHs (into sweat and digestive fluids) and phthalates (into digestive fluids), and in all samples not tested for migration. A repeat experiment in 2017 with the Tiny-TIM model in six rubber granulate samples (three of which were included in the original experiment) confirmed the release of PAHs from rubber granulate into the gastrointestinal tract to be in the order of 10% [24]. The results of the Tiny-TIM model relate to the ‘total amount released’; since substances may still be bound to suspended matter or lipids, which could reduce absorption through the intestinal wall, using the ‘total amount released’ might represent a worst-case scenario for the amount actually available for absorption.

### Risk assessment

Where available for a substance, the migration data were given preference over the content data in calculating the exposure via the oral, dermal and inhalation route. Migration data are considered more relevant for risk assessment than content data, since it is only the fraction of a substance leaching or migrating from the rubber granulate that football players can actually be exposed to. Table 4 specifies the actual values used for the substances selected for risk assessment; in all cases, these concern maximum pitch levels, as worst-case scenario. In the absence of headspace analysis data for the prioritised substances, exposure to vapours was not included for the inhalation route. Hence, for this route only exposure to rubber dust was calculated, with the use of content data as worst-case scenario (no data on migration of substances out of rubber granulate dust into artificial lung fluid are available).

**Table 4:** Maximum concentrations and migration levels (in bold) per pitch used for exposure assessment

Substances	Maximum content/migration values (in mg/kg)		
	Oral	Dermal	Inhalation
Metals			
Cadmium	**0 (<LOD)**	**0.00002**	2.1^a^
Cobalt	**0.002**	**0.00048**	100^b^
Lead	**0.009**	**0.00007**	35^a^
PAHs			
ECHA-8	**1.78**	**0.00396**	19.8
Phthalates^c^			
DEHP	**5.44**	**<LOD**	27.2
DIBP	**0.464**	**<LOD**	2.32
DBP	**0.172**	**<LOD**	0.86
BBP	**0.198**	**<LOD**	0.99
DINP	**12.2**	**<LOD**	61
DCHP	**0.042**	**<LOD**	0.21
Other			
2-MBT	7.6	7.6	7.6
BPA	2.5	2.5	2.5

*LOD* limit of detection

^a^Based on Dutch Milieukeur data (content data from batches of rubber granulate, submitted for certification in the period 2010–2016) [68]

^b^In the absence of Dutch Milieukeur data, a fictitious level of 100 mg/kg was chosen

^c^For dermal exposure calculations, the value of the LOD was taken as a worst case

The exposure calculations yield an estimate of the chemical substance on or in the body that has not yet been absorbed into the bloodstream. The exposure estimates therefore relate to external, not internal, exposure. There was, however, no need to convert the external exposures into internal ones (by adjusting for absorption), as the toxicological reference values for all prioritised substances were external values as well, except for bisphenol A (BPA). For BPA, the external dermal exposure was converted into an internal exposure because the toxicological reference value for the dermal route applies to the absorbed dose.

For each prioritised substance, the combined risk over all routes of exposure was estimated. First, the risk per route was assessed (by comparing, per route, the exposure estimates calculated for the various scenarios to the toxicological reference values), and subsequently these risks were summed. For threshold substances this resulted in a total RCR, for the non-threshold PAHs in an estimation of the additional risk for cancer.

The results show that the oral route is the most important exposure route for PAHs and phthalates in rubber granulate, whereas for BPA it is the dermal route. The contribution of inhalation of rubber dust to the total exposure to the prioritised substances appeared minimal. Supplementary Tables S3-S7 present the detailed results of the exposure and risk assessment.

#### PAHs

**Fig. 1:**
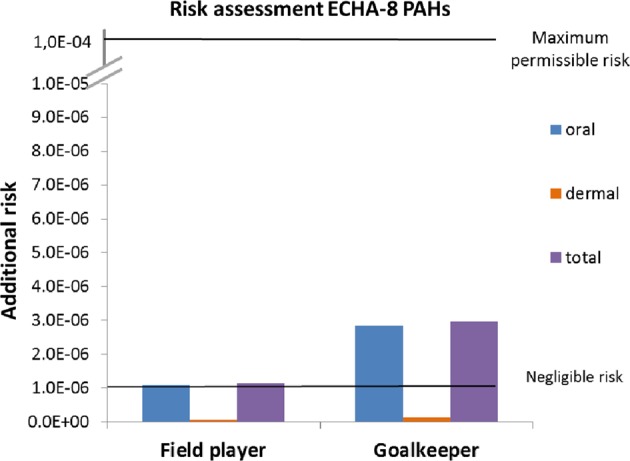
Results of the risk assessment for the PAHs according to the linear extrapolation method; based on maximum migration values. Horizontal lines represent additional cancer risk of one in a million (negligible risk) or one in 10 thousand (maximum permissible risk)

Looking at PAHs in rubber granulate, the dermal route (with a very low migration of 0.02% to sweat) and inhalation route (with a dust exposure of 0.027 ng/m^3^ and vapour concentration of 0.03 ng/m^3^, both well below the air limit value) do not appear to be relevant exposure routes for football players. The oral exposure to PAHs in rubber granulate, taking the ECHA-8 PAHs as marker and assuming that 9% of the PAHs migrate into the gastrointestinal tract, is estimated to be 7.6 × 10^−4^ µg/kg bw/d for a field player and 1.99 × 10^−3^ µg/kg bw/d for a goalkeeper. These oral exposures are associated with additional cancer risks of 1.1 × 10^−6^ (field player) and 2.8 × 10^−6^ (goalkeeper). These risks are at or very slightly above the risk level of 1 × 10^−6^ that is considered negligible in the Netherlands (Fig. 1) (Supplementary Table S3).

#### Phthalates

Under worst-case conditions (estimated exposure based on maximum migration values, not averaged out over the year/lifetime, all training sessions and matches on synthetic turf with rubber granulate), the exposure to various phthalates (DEHP, DIBP, DCHP, DBP, BBP and DINP) in rubber granulate is 2–6 orders of magnitude lower than the toxicological reference values for these phthalates (RCR ≪ 1). Even when accounting for aggregate exposure to multiple phthalates (because of their similar toxicological profile), the total RCR is well below 1. Consequently, the results indicate no cause for concern for exposure to phthalates from rubber granulate, even if the inhalation from vapour (following evaporation from rubber granulate) would additionally contribute to the total exposure (Supplementary Table S4).

#### 2-Mercaptobenzothiazole (2-MBT)/benzothiazoles

The results for 2-MBT are comparable to those of the phthalates. For 2-MBT, the worst-case scenario is even more extreme, because in the absence of migration data the calculations were based on maximum content data. The aggregated exposure to all benzothiazoles also does not appear to result in a health risk. Assuming that they are all converted into the same toxicological metabolite, and comparing the sum total of benzothiazoles with the toxicological reference value for 2-MBT, the total RCR is well below 1 for exposure scenarios 1–4 (data not shown). Thus, the results indicate that exposure to benzothiazoles from rubber granulate is not a cause for concern, even if the inhalation from vapour (following evaporation from rubber granulate) would additionally contribute to the total exposure (Supplementary Table S5).

#### Metals

**Fig. 2:**
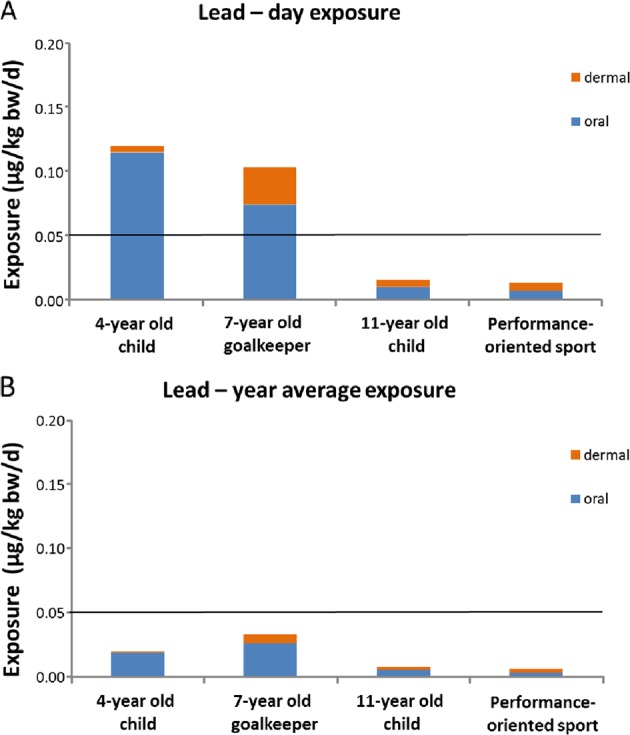
Results of the risk assessment for lead (**A**: day exposure; **B**: year average exposure) based on maximum migration values (oral, dermal). Horizontal line is the maximum tolerable exposure value

Under worst-case conditions (estimated exposure based on maximum migration values, not averaged out over the year/lifetime, all training sessions and matches on synthetic turf with rubber granulate), the risk assessment for the metals cadmium, cobalt and lead in rubber granulate does not reveal a risk for this source of exposure for cadmium and cobalt (all exposure scenarios), nor for lead in scenarios 3 and 4 (Fig. 2a). For children under the age of 11 (i.e., scenarios 1 and 2), the lead exposure under these worst-case conditions is higher than the level that is considered not to present an appreciable health risk (0.05 µg/kg bw/d). The year average exposure for these children, however, remains below that value (Fig. 2b) (Supplementary Table S6). It is to be noted that in the Netherlands, the median exposure to lead via food for children aged 4 (0.88 µg/kg bw/d) and 7 (0.76 µg/kg bw/d) [25] (the ages taken as worst case for scenarios 1 and 2, respectively) is already higher than what is tolerable from a toxicological perspective. Compared with food, the contribution from rubber granulate to lead exposure appears to be fairly limited, in particular when taking into consideration that, in order to achieve the intake as calculated, children would have to ingest 0.2 g of rubber grains during each training/match, and that all of the ‘total amount released’ is indeed available for absorption via the intestinal wall. Further, the dermal exposure was compared with the oral reference value (in the absence of a dermal value), assuming equal absorption via both routes. This is a worst-case assumption, as for metals dermal absorption is generally lower than oral absorption.

Although for all three prioritised metals, the migration was only tested in a small number of samples, the results appear to show that their migration into sweat and saliva/gastric juice/intestinal juice does not exceed the migration limits as set in the Toy Safety Directive [19]. No additional contribution from exposure via inhalation of vapour is expected for the prioritised metals, given their very low vapour pressure.

#### BPA

**Fig. 3:**
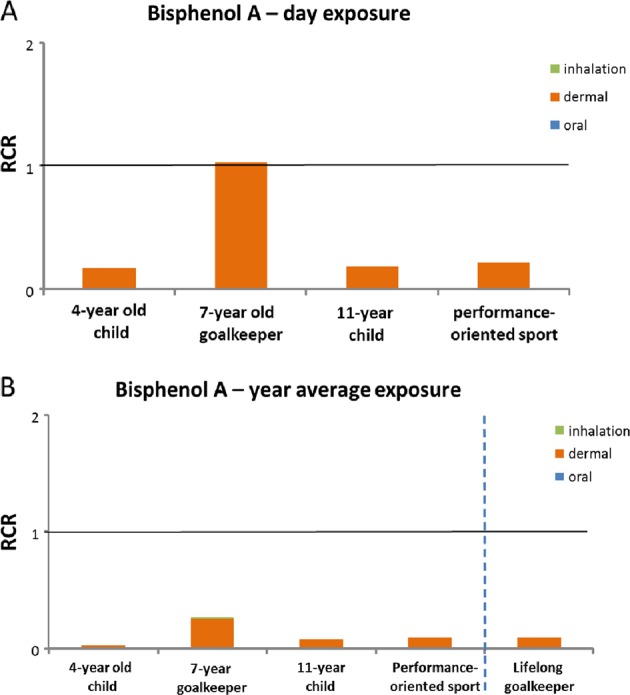
Results of the risk assessment for bisphenol A (**A**: day exposure; **B**: year average exposure and ‘lifelong’ exposure); based on maximum pitch values. Horizontal line is RCR = 1

For BPA, the risk appears almost solely determined by dermal exposure. Under worst-case conditions (estimated exposure based on maximum content values, not averaged out over the year/lifetime, all training sessions and matches on synthetic turf with rubber granulate), there is a possible cause for concern for one of the exposure scenarios, i.e., scenario 2 (Fig. 3a). For this scenario (goalkeeper from 7 years of age, with 7-year-old child as worst case), the RCR equals 1. Following a tiered approach, in the next step the year average and ‘lifelong’ exposures for this scenario were compared with the toxicological reference value for BPA. Using these exposures, for which an adjustment for age is included in the ‘lifelong’ exposure as a person will not be a 7-year-old goalkeeper for his or her entire life, there is no longer a cause for concern (Fig. 3b) (Supplementary Table S7).

The true RCRs for BPA will be lower than currently calculated, since maximum content data had to be used in the absence of migration data for BPA, and it is not likely that 100% of the BPA present in rubber granulate would migrate onto skin. Furthermore, no additional risk is expected via inhalation of vapour given the low vapour pressure of BPA in combination with the reference value for air.

## Discussion

The ambitions formulated for circular economy in various European countries, including the Netherlands, have resulted in a huge increase in investments to recycle old products into raw material for new products. This also applies to car tyres, which have been banned from landfill since 2003 by the European Landfill Directive [1], as a consequence of which recycling was stimulated. The concerns that over the years have arisen about the safety of rubber granulate show that tension may exist between the ambition to increase the reuse of materials on the one hand, and the reintroduction of potentially hazardous substances in new products on the other hand. The research presented here was commissioned following a sudden increase in public concern after it was suggested in a Dutch TV programme that young goalkeepers and football players have an increased risk of getting cancer when playing on synthetic turf football pitches with rubber granulate as infill, due to the presence of carcinogenic substances in the rubber granulate. The main question to answer was therefore whether it is safe to play sports on STP with rubber infill currently in place in the Netherlands. The research was further intended to aid policy makers in deciding on any immediate or future actions. Limit setting for (substances in) rubber granulate was not an objective of the research.

Due to time constraints, a targeted approach was followed, focussing the human health risk assessment on outdoor STP, rubber infill, chemical safety, amateur football players and CMR substances. One hundred STPs were sampled, but as nine pitches consisted entirely or partly of infill material other than SBR, samples of 91 fields were included for analyses. This number gives a representative picture of the rubber infill applied on Dutch STP, being >5% of the STP with SBR infill in the Netherlands, and covering pitches of various ages (1–15 years) and locations all over the country. The chemical analyses showed the presence of a large number of substances in rubber granulate, including various PAHs, phthalates, metals, benzothiazoles and phenols. In contrast, VOCs like benzene, styrene and toluene were nearly absent.

With rubber infill at the moment being considered a mixture in the EU, the results of the sampling study showed that the rubber granulate on all tested pitches complied with the concentration limits set for mixtures of substances in the REACH regulation. However, when rubber infill would be considered an article (like for instance rubber tiles in playgrounds) and the concentration limits for consumer products and toys were to be applied to rubber granulate, then a large number of the samples would not meet these concentration limits. This is almost exclusively due to relatively high concentrations found for some individual PAHs: for BaP, BaA, CHR, BbFA and BeP (i.e., five out of eight PAHs restricted under REACH Annex XVII entry 50) the maximum pitch concentrations exceeded the concentration limits set for their presence in consumer articles (1 mg/kg) and toys (0.5 mg/kg). Aside from the fact that these regulatory limit values do not directly apply to rubber granulate, exceedance of a limit value does not automatically represent a health risk. For that, the substance in question must be released from the rubber granulate to such an extent (by means of leaching/migration) that the toxicological reference value for this substance is exceeded in a specific exposure scenario. For risk assessment, it is therefore not so important to know which substances are present in rubber granulate (content), but rather which of these substances can be released from rubber granulate in significant amounts. This is the reason why the present investigation included migration experiments. Although limited in nature (a relatively low number of measurements were possible in the short timeframe available, for a few groups of substances), they provide an indication as to the fraction of PAHs, phthalates and metals that become actually available for exposure.

As to the results of the content and migration analyses, a comparison with literature data learned that the maximum pitch concentrations measured for the substances in rubber granulate in the present research are generally lower than the maximum concentrations reported in literature on rubber granulate content. The literature data available on migration are very limited but seem to confirm the low to very low migration from substances in rubber granulate into artificial sweat or digestive fluids. A detailed account of the comparison with literature data is presented in the scientific background document [14].

The risk assessment indicated that the oral and dermal routes are the main contributors to the exposure of amateur football players to substances in rubber granulate; the inhalation route appeared not to contribute. No concern was identified for their exposure to the prioritised phthalates, benzothiazoles, BPA and metals in rubber granulate from Dutch STP, not even under worst-case conditions, as the estimated exposures to these substances were below the exposures that would lead to adverse effects on health. The prioritised PAHs appeared to be the substances of the highest concern, but even for this group of substances the measured migration from the Dutch field samples resulted in estimated additional cancer risks that were just above the negligible risk level of one in a million. The results from the present investigation are in concordance with the findings in previous national and international investigations into the health risks of rubber granulate [26–34], and were recently confirmed in the investigation by ECHA [12].

An increased risk of leukaemia and lymphoma, the two cancer types for which specific concern was expressed in the TV broadcast, was not evident from our investigation. Substances known or suspected to be associated with these types of cancer were either not detected in any of the rubber granulate samples (benzene, styrene and 1,3-butadiene), or present in such low concentrations (2-MBT) that no risk can be expected. From our results, it can further be seen that it is primarily the PAHs in rubber granulate that may potentially lead to an increased cancer risk. For PAHs, there is, however, no clear link with leukaemia and lymphoma [35, 36]. Bleyer and Keegan recently concluded that avoidance of STP for fear of increased cancer risk is not warranted. Their study in California, the state in the US with the greatest number of STP with crumb rubber, showed no association between individual-level exposures to such turf fields and malignant lymphoma [37].

### Uncertainties and their impact

The main uncertainties in the risk assessment performed relate to the targeted approach taken, the assumptions and values taken for some exposure parameters, and the toxicological reference values used for some prioritised substances. These are discussed below. In addition, an assessment of the combined health effects of all the substances present in rubber granulate was beyond the scope of the study. This uncertainty is, however, not considered to affect the main conclusions of the risk assessment. The assessment further only examined a single source of exposure to a substance, namely from rubber granulate. As such, the exposure to the prioritised substances was shown not to present a health risk. But as these substances occur in other sources as well, rubber granulate may still contribute to a possible health risk when the total exposure via all sources exceeds the toxicological reference value of a substance. For substances with very low RCRs (e.g., the phthalates and benzothiazoles), there is room for other sources of exposure. For lead, however, the intake via food is already higher than what is tolerable from a toxicological perspective. Exposure via rubber granulate will add to this, but not much, as exposure to lead via rubber granulate is at least 7.5- to 10-fold lower compared with lead ingested via food. Also for PAHs rubber granulate is one of many sources of exposure; others include, for example, exhaust fumes, tyre particulates, cigarette smoke, burned wood (open fire) and meat (barbecue). Compared with food as the most important source of PAHs for the general population (non-smokers), the estimated exposure via rubber granulate (37–98 ng/day versus 1800–4900 ng/day via food for the ECHA-8 PAHs) is marginal. PAHs exposure via food may even be significantly higher when a person eats barbecued meat on a regular basis [38].

#### Targeted approach

The risk assessment was focussed on some prioritised substances (i.e., those present in levels above regulatory limit values ànd having CMR properties), not on all substances present in rubber granulate. Performing a risk assessment for substances present in levels below regulatory limit values was, however, not considered an immediate priority. Although this does not automatically mean that these substances are safe, the idea is that when the concentration of a substance stays below the limit value(s) considered acceptable for that particular substance in products/media other than rubber infill, this concentration should also be acceptable for that substance in rubber infill. Given the results reached for the prioritised substances, it is indeed not expected that the non-prioritised substances would present a health risk. It is acknowledged that the non-prioritised substances may have effects other than CMR. However, the CMR endpoints are generally seen as the most serious for human health, with the reprotox endpoint partly also covering endocrine disruption effects. It is further to be noted that the toxicological reference values for the prioritised substances cover all endpoints of the substance, not only CMR, as these values are based on the most sensitive effect within the toxicological profile of the substance. It may thus be assumed that the toxicological reference values found for the prioritised substances cover all hazardous properties relevant to human health including, but not limited to, CMR. From the result of the ‘general unknown screening’ it further appears that we have not missed CMR substances that were not à priori part of the analysis program.

The targeting on outdoor STP and amateur football players (children/adults) means that indoor STP and very small children playing, professional football players and workers installing or maintaining the STPs were not part of the risk assessment. Whereas indoor STP are not relevant for the Dutch situation (all STP are outdoor), it is noted that indoor STPs were included in the investigation by ECHA. No risk was identified, albeit a recommendation was made to take care of good ventilation in indoor halls, to avoid high concentrations of VOCs, some of which might cause irritation to the respiratory tract, skin and eyes. ECHA’s investigation also included a risk assessment for professional football players and for workers; for both target populations no risks were identified [12].

#### Exposure parameters

In the absence of reliable data for a number of input parameters, values were derived from literature. In the near future, the EPA and OEHHA research into exposure characteristics/scenarios can hopefully help in generating more relevant and realistic input values [8, 10]. For the moment, it is, however, considered that the assumptions made and input values taken for the exposure estimation are rather worst case and therefore err on the side of caution. For instance, the assumption that a single person all his/her life will play every training session and every match on synthetic turf with rubber granulate, will in reality only be true for a small group of people, as natural grass and pitches with other infill material will be played on as well. Also the assumption that children would ingest 0.2 g of rubber grains during each and every training session/match (and adults 0.05 g) is worst case. Too worst case according to ECHA, not only the frequency, but also the amount. ECHA assumed 0.05 g for children (and 0.01 g for adults) in their risk assessment, but still considered this to be an overestimation [12]. It is noted that in the most recent update of EPA’s Exposure Factors Handbook [39], the value of 0.2 g for soil/dust ingestion for children has been refined to 0.09 g (soil only). All in all, the majority of football players will have a much lower exposure than what has conservatively been calculated. This reduces the concern, as the risks calculated are overestimated.

Although migration data are more relevant for risk assessment than content data, it was not possible within the given timeframe to conduct migration experiments for all samples and all prioritised substances. For the prioritised substances without migration data (2-MBT and BPA), the risks calculated are therefore overestimated, as these had to be based on content data. For the prioritised substances with migration data, the results of the migration experiments do not provide a comprehensive overview, due to the limited number of samples tested. The uncertainty as to whether the results can be extrapolated to the samples not tested is, however, somewhat reduced by the findings of a fairly robust relationship between the content and migration level for both the PAHs and the phthalates.

For PAHs, there is some additional uncertainty due to the use of sweat migration data. With PAHs being lipophilic compounds, the migration to non-sweaty skin may have been underestimated, as skin is also somewhat lipohilic in nature. Although a few studies have been reported in literature investigating the migration of PAHs from rubber onto a strong adsorbent or into more lipophilic matrices like Vaseline, massage oil and 20% ethanol [26, 40, 41], these do not allow the determination of the percentage migration. Further, no studies have been reported in which PAH migration from rubber granulate into both sweat and a lipophilic medium have been tested under the same conditions. Hence, the degree of underestimation is unknown. Given, however, the relatively short contact time with rubber granulate when playing football (maximally 2 h), as well as the very small skin surface area that is in direct contact with the rubber grains, the expectation is that dermal absorption will only limitedly contribute to total absorption, even with higher migration. To illustrate this: when for PAHs a 10 times higher migration into a lipophilic matrix is assumed than into sweat (so 0.2%), the resulting additional cancer risks would only be marginally higher (1.7 × 10^−6^ for field player and 4.2 × 10^−6^ for goalkeeper).

#### Toxicological reference values

As explained, we applied toxicological reference values as derived/used by international scientific committees or organisations in their risk assessment of these substances. The basis for the toxicological reference values might not have included the latest information or insights on the substances. For instance, four phthalates (DEHP, DBP, BBP, DIBP) were recently identified as endocrine disruptors to human health [42]. This could mean that their toxicological reference values, which are now based on reprotox, may not be sufficiently protective for all effects induced by endocrine disruption. It is noted though that the present risk assessment resulted in RCRs very much lower than one, even for combined phthalate exposure. The large safety margin leaves some room for a lower toxicological reference value.

BPA was also recently identified as endocrine disruptor to human health [43] and some studies indicate that immunotoxic effects of BPA could possibly warrant a lower toxicological reference value than currently in place, may be even a factor 10 lower [44]. Although up till now no such adjustment has formally taken place, a lowering of the toxicological reference value by, e.g., 10-fold would result in the year average RCR becoming >1 for the goalkeeper scenario (scenario 2). As noted before, this RCR is worst case, not only because of the conservative nature of the exposure estimation itself, but also because for BPA content rather than migration data had to be used. For a more realistic risk assessment, BPA needs to be tested for skin migration. Subsequently using the migration data as input for the exposure estimation may give better insight in whether or not there actually would be a concern for this scenario.

For cobalt, the toxicological reference values used relate to threshold effects. However, when recently evaluating five water-soluble cobalt salts under REACH, it was concluded that at present, due to lack of identified thresholds and due to remaining uncertainties regarding the mechanisms involved, their risk for inducing local tumours via inhalation needs to be assessed using a non-threshold approach [45]. As a consequence, the Tolerable Concentration in Air used for cobalt may not be sufficiently protective for the inhalation route. Any lowering of the toxicological reference value for this exposure route is, however, not expected to have a major impact on the conclusions drawn for cobalt, since for metals the inhalation route contributes very little to the total exposure.

The basis of the toxicological reference value for the PAHs in rubber granulate is a mouse carcinogenicity study with a coal tar mixture [46]. This introduces an uncertainty, due to differences in content and perhaps potency between the tested coal tar mixture and the PAH mixture present in rubber granulate. It is, however, not clear what the exact effect of this difference is and whether it results in an underestimation or overestimation of the risk. A further, more general, complication of using animal carcinogenicity studies for risk assessment for young children is that standard carcinogenicity studies do not inform on ‘early-life exposure’: exposure in these studies starts when the animals are around 6–8 weeks of age, corresponding to the period of adolescence in humans. In the US, EPA and OEHHA apply an ‘age-dependent adjustment factor’ (ADAF) to calculate the cancer risk when using linear extrapolation based on a standard animal study. The value of the ADAF should preferably be determined based on substance-specific information; otherwise it is, by default, 10 for the 0–2 years old group and 3 for the 2–16 years old group. The default ADAF for people aged 16 and up is 1 [47, 48]. In Europe and the Netherlands, there are at the moment no such rules under any regulatory framework; in line with the standard practice, we did therefore not apply an additional factor in the risk assessment of children aged 4–15 to account for any intraspecies differences as a consequence of ‘early-life exposure’. Further research and discussion in an international context is, however, highly recommended, in order to reach scientific consensus and harmonisation on how to deal with this important topic in cancer risk assessment. It is noted that for the present investigation, would an ADAF of 3 have been applied (like was done by Ginsberg and Toal in their risk assessment of PAHs in rubber crumb infill on artificial turf fields in Connecticut [32], and by EPA in their recent toxicological review of BaP [49], the risks would be higher than currently calculated, but still well below the maximum permissible risk level of 1 × 10^−4^ (i.e., the level used as tool in the Netherlands to determine whether drastic measures are needed to reduce the risk). In fact, when taking into account all identified uncertainties in the risk assessment performed for PAHs (some of which clearly tend to overestimate the risks), it is not expected that the additional cancer risks for PAHs in rubber granulate will be higher than what has currently been estimated (around the negligible risk of one in a million).

## Conclusion

Our research showed the presence of a number of CMR substances in the rubber granulate on Dutch pitches, i.e., several PAHs, phthalates and benzothiazoles, BPA and metals like cadmium, cobalt and lead. From a regulatory perspective, it can be concluded that for all tested pitches the rubber granulate complies with the concentration limits set for mixtures of substances in Europe. From a risk assessment perspective, it can be concluded that exposure to these CMR substances presents no appreciable health risk, given the concentrations in which they occurred in or migrated from the rubber granulate currently in place on a representative number of STP in the Netherlands. PAHs appeared to be the substances of the highest concern, but even for this group of substances, the concentrations in which they are present do not result in additional cancer risks above the negligible risk level of one in a million. This is supported by the recent investigation by ECHA, in which it was concluded that the concern for lifetime cancer risk is very low given the concentrations of PAHs typically measured in European STP with rubber infill.

A positive answer could therefore be given to the main question raised by the Minister: based on the current evidence available, it is considered safe to play sports on STP with the rubber infill in place in the Netherlands. No immediate action was thus required. It was recommended though to review the conclusions when the results of the ongoing, large-scale studies in the US become available. Further, it was recognised that, should the rubber granulate have contained concentrations of PAHs as high as the European concentration limits for mixtures, safe use might not be guaranteed. To ensure therefore the supply of rubber granulate with only very low concentrations of hazardous substances (PAHs in particular) and thus the safety for people playing sports, it was recommended to set regulatory limit values specifically for (substances in) rubber granulate. Since the publication of our report, this recommendation has resulted in a joint effort of Dutch authorities and ECHA to elaborate a proposal for such limit values [50].

## Supplementary information

Supplementary Tables
