# Supplementary material for: Synthetic turf pitches with rubber granulate infill: are there health risks for people playing sports on such pitches?
Source: J Expo Sci Environ Epidemiol. 2018 Dec 19;30(3):567–84. doi: 10.1038/s41370-018-0106-1 (PMC7181390; doi:10.1038/s41370-018-0106-1)
Supplement: Supplementary file 1 — Supplementary Tables [file 41370_2018_106_MOESM1_ESM.docx]

Table S1. Comparison of concentrations of substances in rubber granulate with the concentration limit for mixtures and with other regulatory limits. For metals these concern maximum concentrations as reported in literature, in absence of metal content data for the samples. All other substances reported have been detected in at least 5% of the samples analysed for these substances, presenting per substance the median (P50) and maximum pitch concentrations. Where the maximum (pitch) concentration exceeds the limit value, this limit value is marked in red. For all reported substances the CMR classification (the 2^nd^ prioritisation criterion) is also indicated.

| **Substance** | **No. of samples analysed** | **% samples**  **> LOD** | **Concentration (mg/kg dry matter)** | | **Concentration limit for mixtures**  **(mg/kg)** | **Other limit values (mg/kg)** | | | | | **CMR classify-cation** |
| --- | --- | --- | --- | --- | --- | --- | --- | --- | --- | --- | --- |
|  |  |  | **P50** | **Maxi-mum** |  | **Consumer articles** | **Toys** | **Toys** ^1^ | **Building materials** ^2^ | **Soil**  **(residential quality)** |  |
|  |  |  |  |  | **[REACH Annex XVII**  **entry 28-30]** | **[REACH Annex XVII**  **(entry nr)]** | | **[Toy Safety Directive]** | **[Soil Quality Decree]** | | **[C&L Inventory]** |
| **PAHs** |  |  |  |  |  |  |  |  |  |  |  |
| anthracene | 546 | 5 | < 0.5 | 1.1 | - | - | - | - | 10 | - | Carc. 2 |
| benzo[a]anthracene | 546 | 27 | < 0.5 | 2.2 | 1000 | **1**  (50) | **0.5** (50) | - | 40 | - | Carc. 1B |
| benzo[a]pyrene | 546 | 25 | < 0.5 | 2.2 | 100  1000  3000 | **1**  (50) | **0.5** (50) | - | 10 | - | Carc. 1B  Muta. 1B  Repr. 1B |
| benzo[b]fluoranthene ^3^ | 546 | 48 | < 0.5 | 3.0 | 1000 | **1**  (50) | **0.5** (50) | - | - | - | Carc. 1B |
| benzo[c]fluorene | 7 | 43 | 0.2 | 0.7 | - | - | - | - | - | - | - |
| benzo[e]pyrene | 7  (🡪 546) ^4^ | 57 | 2.8 | 7.8 | 1000 | **1**  (50) | **0.5** (50) | - | - | - | Carc. 1B |
| benzo[g,h,i]perylene | 546 | 62 | 4.1 | 7.7 | - | - | - | - | 40 | - | - |
| chrysene | 546 | 57 | 1.3 | 3.5 | 1000 | **1**  (50) | **0.5** (50) | - | 10 | - | Carc. 1B  Muta. 2 |
| cyclopenta[c,d]pyrene | 7 | 100 | 1.5 | 2.5 | - | - | - | - | - | - | - |
| phenanthrene | 546 | 38 | < 0.5 | 7.1 | - | - | - | - | 20 | - | Carc. 2 |
| fluoranthene | 546 | 93 | 3.4 | 20.3 | - | - | - | - | 35 | - | - |
| pyrene | 546 | 98 | 7.5 | 28.7 | - | - | - | - | - | - | - |
| ECHA-8 ^5^ | 546 |  | 5.8 | 19.8 |  |  |  |  |  |  |  |
| **Phthalates** |  |  |  |  |  |  |  |  |  |  |  |
| di-2-ethylhexyl phthalate | 546 | 100 | 7.6 | 27.2 | 3000 | 1000 (51/52) | - | - | - | **8.3** | Repr. 1B |
| diisononyl phthalate | 42 | 77 | 35 | 61 | - | 1000 (51/52) | - | - | - | - | - |
| bis (2-ethylhexyl) adipate | 42 | 63 | 0.3 | 1.1 | - | - | - | - | - | - | - |
| diisobutyl phthalate | 546 | 17 | < 0.5 | 2.3 | 3000 | - | - | - | - | **1.3** | Repr. 1B |
| dicyclohexyl phthalate | 42 | 47 | 0.1 | 0.2 | 3000 | - | - | - | - | - | Repr. 1B |
| di-n-nonyl phthalate | 42 | 37 | 0.5 | 0.8 | - | - | - | - | - | - | - |
| diphenyl phthalate | 42 | 7 | < 0.1 | 0.11 | - | - | - | - | - | - | - |
| **Benzothiazoles** |  |  |  |  |  |  |  |  |  |  |  |
| benzothiazole | 7 | 100 | 2.7 | 6.3 | - | - | - | - | - | - | - |
| 2-hydroxybenzothiazole | 7 | 100 | 1.6 | 13.8 | - | - | - | - | - | - | - |
| 2-mercaptobenzothiazole | 7 | 100 | 2.6 | 7.6 | - | - | - | - | - | - | Carc. 1B |
| 2-methoxybenzothiazole | 7 | 100 | 2.6 | 10.2 | - | - | - | - | - | - | - |
| 2-aminobenzothiazole | 7 | 100 | 0.10 | 0.38 | - | - | - | - | - | - | - |
| N-cyclohexyl-1,3-benzothiazole-2-amine | 7 | 100 | 1.5 | 3.9 | - | - | - | - | - | - | - |
| 2,2-dithiobis(benzothiazole) | 7 | 71 | 0.19 | 0.33 | - | - | - | - | - | - | - |
| N-cyclohexyl-2-benzothiazole sulphenamide | 7 | 43 | < 0.02 | 0.04 | - | - | - | - | - | - | - |
| **Phenols** |  |  |  |  |  |  |  |  |  |  |  |
| 4-t-octylphenol | 7 | 100 | 4.8 | 22.4 | - | - | - | - | - | - | - |
| bisphenol A | 7 | 100 | 0.5 | 2.5 | 3000 | - | - | **0.1** mg/L | - | - | Repr 1B |
| **PCBs** ^6^ |  |  |  |  |  |  |  |  |  |  |  |
| PCB28 | 7 | 14 | < 0.005 | 0.015 | - | - | - | - | - | - | - |
| PCB101 | 7 | 29 | < 0.005 | 0.020 | - | - | - | - | - | - | - |
| PCB153 | 7 | 29 | < 0.005 | 0.030 | - | - | - | - | - | - | - |
| PCB138 | 7 | 14 | < 0.005 | 0.014 | - | - | - | - | - | - | - |
| PCB180 | 7 | 14 | < 0.005 | 0.012 | - | - | - | - | - | - | - |
| total PCBs |  | 29 | < 0.035 | 0.074 | - | - | - | - | 0.5 | **0.04** | - |
| **Metals** |  |  |  |  |  |  |  |  |  |  |  |
| cadmium |  |  |  | 2.1 | 1000 | 100 or 1000  (23) | - | **1.3** | [0.04] | **0.4** | Carc. 1B Muta. 2  Repr. 2 |
| cobalt |  |  |  | 234 | - | - | - | **10.5** | [0.54] | **123** | Carc. 1B Muta. 2  Repr. 1B |
| lead |  |  |  | 35 | 300 or  3000 | 500  (63) | - | **13.5** | [2.3] | 119 | Repr. 1A |
| zinc |  |  |  | 17700 | - | - | - | **3750** | [4.5] | **138** | - |

^1^ The limit values for toys are migration limits. Comparison of the maximum content value with these migration limits is worst case.

^2^ For metals, the limit values do not concern maximum content limits (like for the other substances), but maximum emission limits, based on leaching. The comparison with maximum content values is therefore not appropriate.

^3^ Includes benzo[j]fluoranthene (overlapping peaks in the chromatogram)

^4^ Because of the strong correlation with chrysene (r^2^=0.98), estimates were made for all pitches using the formula [BeP] = 2.2467 x [CHR]

^5^ ECHA-8 concerns the eight PAHs in REACH Annex XVII, entry 50 (benzo[a]pyrene, benzo[e]pyrene, benzo[a]anthracene, chrysene, benzo[b]fluoranthene, benzo[j]fluoranthene, benzo[k]fluoranthene and dibenzo[a,h]anthracene)

^6^ PCB28, PCB101, PCB138, PCB153 and PCB180 were the only PCBs found. All five are ‘non-dioxin like’ PCBs, having no CMR classification.

(LOD = limit of detection; PAHs = polycyclic aromatic hydrocarbons; PCBs = polychlorinated biphenyls)

Table S2. Migration/leaching of substances from rubber granulate into gastric/intestinal juices (after four hours of in vitro digestion at 37°C), artificial sweat (after two hours of exposure at 37°C) and water (after 24 hours of exposure at room temperature). Reported are the maximum values as found for a pitch.

| **Substance** | **Migration into artificial gastric/intestinal juices ***  **(in µg/g)** | **Migration into artificial sweat**  **(in ng/g)** | | **Leaching into water**  **(in mg/kg)** | |
| --- | --- | --- | --- | --- | --- |
| **PAHs** | **n=5 samples** ^#^ | **n=7 samples** ^$^ | **n > LOD** | n/a |  |
| acenaphthene | 0.02 | <0.5 | 0 |  |  |
| acenaphthylene | < LOD | <0.4 | 0 |  |  |
| anthracene | < LOD | <0.3 | 0 |  |  |
| benzo[a]anthracene | 0.01 | <0.3 | 0 |  |  |
| benzo[a]pyrene | 0.03 | <0.4 | 0 |  |  |
| benzo[b]fluoranthene | 0.05 | <0.3 | 0 |  |  |
| benzo[g,h,i]perylene | 0.29 | 1.02 | 5 |  |  |
| benzo[k]fluoranthene | < LOD | <0.3 | 0 |  |  |
| chrysene | 0.15 | 0.31 | 2 |  |  |
| dibenzo[a,h]anthracene | 0.02 | 1.08 | 1 |  |  |
| phenanthrene | 0.13 | <0.3 | 0 |  |  |
| fluoranthene | 1.02 | 0.61 | 3 |  |  |
| fluorene | < LOD | <0.4 | 0 |  |  |
| indeno[1,2,3-cd]pyrene | 0.03 | <0.5 | 0 |  |  |
| naphthalene | 0.37 | 0.39 | 3 |  |  |
| pyrene | 1.13 | 1.76 | 4 |  |  |
| benzo[e]pyrene ^1^ | 0.34 | 0.70 | 2 |  |  |
| **Phthalates** | **n=5 samples** ^#^ | **n=7 samples** |  | n/a |  |
| butylbenzyl phthalate | 0.29 | < LOD |  |  |  |
| dibutyl phthalate | 0.08 | < LOD |  |  |  |
| dicyclohexyl phthalate | 0.27 | < LOD |  |  |  |
| bis (2-ethylhexyl) adipate | < LOD | < LOD |  |  |  |
| bis (2-n-ethylhexyl) phthalate | 1.84 | < LOD |  |  |  |
| diethyl phthalate | 0.26 | < LOD |  |  |  |
| diisobutyl phthalate | 0.18 | < LOD |  |  |  |
| diisodecyl phthalate | 0.28 | < LOD |  |  |  |
| diisononyl phthalate | < LOD | < LOD |  |  |  |
| dimethyl phthalate | 0.05 | < LOD |  |  |  |
| di-n-nonyl phthalate | 0.06 | < LOD |  |  |  |
| di-n-octyl phthalate | < LOD | < LOD |  |  |  |
| diphenyl phthalate | 0.09 | < LOD |  |  |  |
| **Metals** | **n=2 samples** | **n=7 samples** | **n > LOD** | **n = 546 samples** | **n > LOD** |
| antimony | < LOD |  |  | < LOD | 0 |
| arsenic | < LOD |  |  | < LOD | 0 |
| barium | 6 |  |  | 0.23 | 89 |
| cadmium | < LOD | 20 | 1 | < LOD | 0 |
| chromium | 1 |  |  | 0.018 | 16 |
| cobalt | 2 | 480 | 7 | 0.38 | 363 |
| copper | 78 |  |  | 0.87 | 425 |
| mercury | < LOD |  |  | 0.0006 | 2 |
| lead | 9 | 70 | 7 | 0.10 | 1 |
| molybdenum | < LOD |  |  | < LOD | 0 |
| nickel | 2 |  |  | 0.11 | 2 |
| selenium | 1 |  |  | 0.041 | 3 |
| tin | < LOD |  |  | < LOD | 0 |
| titanium | 1 |  |  | 0.18 | 12 |
| vanadium | < LOD |  |  | < LOD | 0 |
| zinc | 419 |  |  | 129 | 546 |

^1^ Estimated from 2.2467 x [chrysene]

^*^ Total amount released, which is the sum total of the amount in filtrate and in the liquid part of the residue. This represents a worst case scenario, given that the substances may still be bound to suspended matter or lipids, which could reduce absorption through the intestinal wall.

^#^ In the five samples, there appears to be a reasonably robust correlation between the content of the various detectable PAHs and phthalates and the amount of these PAHs and phthalates released into the gastrointestinal tract (approximately 9 and 20%, respectively). These fractions were therefore used to estimate the migration for all PAHs and phthalates, and in all samples not tested for migration, resulting in the following maximum migration levels:

|  | Content  (maximum pitch value) | Migration level (maximum) |
| --- | --- | --- |
| **PAHs** |  |  |
| ECHA-8 | 19.8 mg/kg * 9% = | 1.78 mg/kg |
| **Phthalates** |  |  |
| DBP | 0.86 mg/kg * 20% = | 0.172 mg/kg |
| BBP | 0.99 mg/kg * 20% = | 0.198 mg/kg |
| DEHP | 27.2 mg/kg * 20% = | 5.44 mg/kg |
| DIBP | 2.32 mg/kg * 20% = | 0.464 mg/kg |
| DINP | 61 mg/kg * 20% = | 12.2 mg/kg |
| DCHP | 0.21 mg/kg * 20% = | 0.042 mg/kg |

For metals this cannot be done, given the very small number of samples and the lack of content data.

^$^ In the seven samples a fairly constant relationship was observed between the total concentration of the detectable PAHs in the rubber granulate and their migration into sweat (approximately 0.02%). This percentage was therefore used to estimate the migration into sweat for all PAHs, and in all samples not tested for migration, resulting in the following maximum migration levels:

|  | Content  (maximum pitch value) | Migration level (maximum) |
| --- | --- | --- |
| **PAHs** |  |  |
| ECHA-8 | 19.8 mg/kg * 0.02% = | 3.96 µg/kg |

(LOD = limit of detection; n/a = not analysed; PAHs = polycyclic aromatic hydrocarbons; ECHA-8 = eight PAHs in REACH Annex XVII, entry 50 (benzo[a]pyrene, benzo[e]pyrene, benzo[a]anthracene, chrysene, benzo[b]fluoranthene, benzo[j]fluoranthene, benzo[k]fluoranthene and dibenzo[a,h]anthracene))

Table S3. Results of the risk assessment for the ECHA-8 PAHs for exposure scenario 5 (‘lifelong’ exposure) *; according to the linear extrapolation method and based on maximum migration values

| **ECHA-8** |  | **max. migration level (mg/kg rubber granulate)** | **lifelong exposure**  **(µg/kg bw/d)** | **BMDL_10_ (µg/kg bw/d)** | **Additional risk per µg/kg bw/d** | **Additional risk** |
| --- | --- | --- | --- | --- | --- | --- |
| **Field player** | **oral** | 1.78 | 7.60E-04 | 490 | 1.43E-03 | 1.09E-06 |
|  | **dermal** | 0.00396 | 6.57E-05 | 740 | 9.46E-04 | 6.21E-08 |
|  | ***total*** |  |  |  |  | *1.15E-06* |
| **Goalkeeper** | **oral** | 1.78 | 1.99E-03 | 490 | 1.43E-03 | 2.84E-06 |
|  | **dermal** | 0.00396 | 1.44E-04 | 740 | 9.46E-04 | 1.36E-07 |
|  | ***total*** |  |  |  |  | *2.98E-06* |

* Inhalation exposure is not included, because the toxicological reference value is based on a different marker and carcinogenic effect than the oral and dermal reference values. The additional cancer risk associated with inhalation exposure can therefore not be added. For information, the calculated inhalation exposure to PAHs via particulate matter (0.027 ng/m^3^) and vapour (0.03 ng/m^3^), both based on BaP as a marker for the PAHs present in rubber granulate, is well below the limit value for BaP in air (1 ng/m^3^).

(BMDL_10_ = 95 percent lower confidence level of the dosage resulting in a 10% additional cancer risk in laboratory animals upon lifelong exposure)

Table S4. Results of the risk assessment for the phthalates for exposure scenarios 1, 2, 3 and 4; based on maximum migration (oral, dermal) or pitch values (inhalation)

**Scenario 1:** **Field player aged 4 to 11 years (recreational)** (with 4-year old child as worst case)

|  | **Oral** | | | **Dermal *** | | | **Inhalation** | | | **Total** |
| --- | --- | --- | --- | --- | --- | --- | --- | --- | --- | --- |
| **4-year old child** | **Exposure (µg/kg bw/d)** | **DNEL (µg/kg bw/d)** | **RCR** | **Exposure (µg/kg bw/d)** | **DNEL (µg/kg bw/d)** | **RCR** | **Exposure (µg/m^3^)** | **DNEL (µg/m^3^)** | **RCR** | **RCR** |
| DBP | 2.19E-03 | 6.7 | 3.27E-04 | 1.08E-05 | 70 | 1.55E-07 | 1.03E-05 | 20 | 5.16E-07 | 3.28E-04 |
| BBP | 2.52E-03 | 500 | 5.04E-06 | 1.08E-05 | 10000 | 1.08E-09 | 1.19E-05 | 1700 | 6.99E-09 | 5.05E-06 |
| DEHP | 6.93E-02 | 34 | 2.04E-03 | 1.06E-04 | 672 | 1.58E-07 | 3.26E-04 | 120 | 2.72E-06 | 2.04E-03 |
| DIBP | 5.91E-03 | 8.3 | 7.12E-04 | 1.08E-05 | 80 | 1.35E-07 | 2.78E-05 | 25 | 1.11E-06 | 7.13E-04 |
| DINP | 0.1554 | 250 | 0.000622 | 1.06E-03 | 6250 | 1.70E-07 | 7.32E-04 | 870 | 8.41E-07 | 6.23E-04 |
| DCHP | 5.35E-04 | 180 | 2.97E-06 | 1.08E-05 | 1800 | 6.02E-09 | 2.52E-06 | 630 | 4.00E-09 | 2.98E-06 |
| *Sum* |  |  | *3.71E-03* |  |  | *6.25E-07* |  |  | *5.20E-06* | *3.71E-03* |

**Scenario 2:** **Goalkeeper from 7 years of age** (with 7-year old child as worst case)

|  | **Oral** | | | **Dermal *** | | | **Inhalation** | | | **Total** |
| --- | --- | --- | --- | --- | --- | --- | --- | --- | --- | --- |
| **7-year old goal-keeper** | **Exposure (µg/kg bw/d)** | **DNEL (µg/kg bw/d)** | **RCR** | **Exposure (µg/kg bw/d)** | **DNEL (µg/kg bw/d)** | **RCR** | **Exposure (µg/m^3^)** | **DNEL (µg/m^3^)** | **RCR** | **RCR** |
| DBP | 1.42E-03 | 6.7 | 2.11E-04 | 7.00E-05 | 70 | 9.99E-07 | 1.03E-05 | 20 | 5.16E-07 | 2.13E-04 |
| BBP | 1.63E-03 | 500 | 3.26E-06 | 7.00E-05 | 10000 | 7.00E-09 | 1.19E-05 | 1700 | 6.99E-09 | 3.27E-06 |
| DEHP | 4.48E-02 | 34 | 1.32E-03 | 6.87E-04 | 672 | 1.02E-06 | 3.26E-04 | 120 | 2.72E-06 | 1.32E-03 |
| DIBP | 3.82E-03 | 8.3 | 4.60E-04 | 7.00E-05 | 80 | 8.74E-07 | 2.78E-05 | 25 | 1.11E-06 | 4.62E-04 |
| DINP | 0.1004 | 250 | 0.0004016 | 6.86E-03 | 6250 | 1.10E-06 | 7.32E-04 | 870 | 8.41E-07 | 4.04E-04 |
| DCHP | 3.46E-04 | 180 | 1.92E-06 | 7.00E-05 | 1800 | 3.89E-08 | 2.52E-06 | 630 | 4.00E-09 | 1.96E-06 |
| *Sum* |  |  | *2.40E-03* |  |  | *4.04E-06* |  |  | *5.20E-06* | *2.40E-03* |

Table S4 – continued

**Scenario 3:** **Field player aged 11 to 18 years (performance-oriented)** (with 11-year old child as worst case)

|  | **Oral** | | | **Dermal *** | | | **Inhalation** | | | **Total** |
| --- | --- | --- | --- | --- | --- | --- | --- | --- | --- | --- |
| **11-year old child** | **Exposure (µg/kg bw/d)** | **DNEL (µg/kg bw/d)** | **RCR** | **Exposure (µg/kg bw/d)** | **DNEL (µg/kg bw/d)** | **RCR** | **Exposure (µg/m^3^)** | **DNEL (µg/m^3^)** | **RCR** | **RCR** |
| DBP | 1.92E-04 | 6.7 | 2.87E-05 | 1.25E-05 | 70 | 1.79E-07 | 1.03E-05 | 20 | 5.16E-07 | 2.93E-05 |
| BBP | 2.21E-04 | 500 | 4.42E-07 | 1.25E-05 | 10000 | 1.25E-09 | 1.19E-05 | 1700 | 6.99E-09 | 4.50E-07 |
| DEHP | 6.07E-03 | 34 | 1.79E-04 | 1.23E-04 | 672 | 1.83E-07 | 3.26E-04 | 120 | 2.72E-06 | 1.81E-04 |
| DIBP | 5.18E-04 | 8.3 | 6.24E-05 | 1.25E-05 | 80 | 1.57E-07 | 2.78E-05 | 25 | 1.11E-06 | 6.37E-05 |
| DINP | 0.01362 | 250 | 5.45E-05 | 1.23E-03 | 6250 | 1.96E-07 | 7.32E-04 | 870 | 8.41E-07 | 5.55E-05 |
| DCHP | 4.69E-05 | 180 | 2.60E-07 | 1.25E-05 | 1800 | 6.96E-09 | 2.52E-06 | 630 | 4.00E-09 | 2.71E-07 |
| *Sum* |  |  | *3.25E-04* |  |  | *7.23E-07* |  |  | *5.20E-06* | *3.31E-04* |

**Scenario 4:** **Field player aged 18 to 35 years (performance-oriented)**

|  | **Oral** | | | **Dermal *** | | | **Inhalation** | | | **Total** |
| --- | --- | --- | --- | --- | --- | --- | --- | --- | --- | --- |
|  | **Exposure (µg/kg bw/d)** | **DNEL (µg/kg bw/d)** | **RCR** | **Exposure (µg/kg bw/d)** | **DNEL (µg/kg bw/d)** | **RCR** | **Exposure (µg/m^3^)** | **DNEL (µg/m^3^)** | **RCR** | **RCR** |
| DBP | 1.25E-04 | 6.7 | 1.87E-05 | 1.48E-05 | 70 | 2.12E-07 | 1.03E-05 | 20 | 5.16E-07 | 1.94E-05 |
| BBP | 1.44E-04 | 500 | 2.88E-07 | 1.48E-05 | 10000 | 1.48E-09 | 1.19E-05 | 1700 | 6.99E-09 | 2.96E-07 |
| DEHP | 3.95E-03 | 34 | 1.16E-04 | 1.46E-04 | 672 | 2.17E-07 | 3.32E-04 | 120 | 2.04E-06 | 1.19E-04 |
| DIBP | 3.37E-04 | 8.3 | 4.06E-05 | 1.48E-05 | 80 | 1.85E-07 | 2.78E-05 | 25 | 1.11E-06 | 4.19E-05 |
| DINP | 0.008866 | 250 | 3.55E-05 | 1.45E-03 | 6250 | 2.33E-07 | 7.32E-04 | 870 | 6.31E-07 | 3.63E-05 |
| DCHP | 3.05E-05 | 180 | 1.70E-07 | 1.48E-05 | 1800 | 8.24E-09 | 2.52E-06 | 630 | 4.00E-09 | 1.82E-07 |
| *Sum* |  |  | *2.11E-04* |  |  | *8.56E-07* |  |  | *4.31E-06* | *2.17E-04* |

* No phthalates were found above the limit of detection in artificial sweat. The limit of detection was taken as the worst case in calculating dermal exposure.

(DNEL = Derived No-Effect level; RCR = Risk Characterisation Ratio)

Table S5. Results of the risk assessment for 2-mercaptobenzothiazole (2-MBT) for exposure scenarios 1, 2, 3 and 4; based on maximum pitch values

|  |  | **Oral** | | | **Dermal** | | | **Inhalation** | | | **Total** |
| --- | --- | --- | --- | --- | --- | --- | --- | --- | --- | --- | --- |
| **Scenario** * | **max level rubber granulate (mg/kg)** | **Exposure (µg/kg bw/d)** | **DNEL (µg/kg bw/d)** | **RCR** | **Exposure (µg/kg bw/d)** | **DNEL (µg/kg bw/d)** | **RCR** | **Exposure (µg/m^3^)** | **DNEL (µg/m^3^)** | **RCR** | **RCR** |
| **1 (4-yr old)** | 7.6 | 0.10 | 310 | 3.12E-04 | 0.48 | 940 | 5.15E-04 | 0.000091 | 1090 | 8.37E-08 | 8.27E-04 |
| **2 (7-yr old)** | 7.6 | 0.06 | 310 | 2.02E-04 | 3.13 | 940 | 3.33E-03 | 0.000091 | 1090 | 8.37E-08 | 3.53E-03 |
| **3 (11-yr old)** | 7.6 | 0.01 | 310 | 2.74E-05 | 0.56 | 940 | 5.96E-04 | 0.000091 | 1090 | 8.37E-08 | 6.23E-04 |
| **4 (adult)** | 7.6 | 0.01 | 310 | 1.78E-05 | 0.66 | 940 | 7.05E-04 | 0.000091 | 1090 | 8.37E-08 | 7.23E-04 |

* Scenario 1: Field player aged 4 to 11 years (recreational) (with 4-year old child as worst case)

Scenario 2: Goalkeeper from 7 years of age (with 7-year old child as worst case)

Scenario 3: Field player aged 11 to 18 years (performance-oriented) (with 11-year old child as worst case)

Scenario 4: Field player aged 18 to 35 years (performance-oriented)

(DNEL = Derived No-Effect level; RCR = Risk Characterisation Ratio)

Table S6. Results of the risk assessment for cadmium, cobalt and lead for exposure scenarios 1, 2, 3 and 4; based on maximum migration (oral, dermal) or content values (inhalation). For lead also the year average oral and dermal exposures are given (in italics).

**Scenario 1:** **Field player aged 4 to 11 years (recreational)** (with 4-year old child as worst case)

|  | **Oral** | | | **Dermal *** | | | **Inhalation ^#^** | | | **Total** |
| --- | --- | --- | --- | --- | --- | --- | --- | --- | --- | --- |
| **4-year old child** | **Exposure (µg/kg bw/d)** | **TDI^$^ (µg/kg bw/d)** | **RCR** | **Exposure (µg/kg bw/d)** | **TDI (µg/kg bw/d)** | **RCR** | **Exposure (µg/m^3^)** | **air limit (µg/m^3^)** | **RCR** | **RCR** |
| Cadmium | 0 | 0.36 | 0 | 0.001 | 0.36 | 0.004 | 2.52E-05 | 0.005 | 5.04E-03 | 8.58E-03 |
| Cobalt | 0.025 | 1.4 | 0.018 | 0.031 | 1.4 | 0.022 | 1.20E-03 | 0.5 | 2.40E-03 | 0.042 |
| Lead | 0.115 | 0.05 | 2.293 | 0.004 | 0.05 | 0.089 | 4.20E-04 | 0.5 | 8.40E-04 | 2.383 |
|  | *0.019* | *0.05* | *0.382* | *0.001* | *0.05* | *0.015* |  |  |  |  |

**Scenario 2:** **Goalkeeper from 7 years of age** (with 7-year old child as worst case)

|  | **Oral** | | | **Dermal *** | | | **Inhalation ^#^** | | | **Total** |
| --- | --- | --- | --- | --- | --- | --- | --- | --- | --- | --- |
| **7-year old goalkeeper** | **Exposure (µg/kg bw/d)** | **TDI^$^ (µg/kg bw/d)** | **RCR** | **Exposure (µg/kg bw/d)** | **TDI (µg/kg bw/d)** | **RCR** | **Exposure (µg/m^3^)** | **air limit (µg/m^3^)** | **RCR** | **RCR** |
| Cadmium | 0 | 0.36 | 0 | 0.008 | 0.36 | 0.023 | 2.52E-05 | 0.005 | 5.04E-03 | 0.028 |
| Cobalt | 0.016 | 1.4 | 0.012 | 0.198 | 1.4 | 0.141 | 1.20E-03 | 0.5 | 2.40E-03 | 0.155 |
| Lead | 0.074 | 0.05 | 1.481 | 0.029 | 0.05 | 0.576 | 4.20E-04 | 0.5 | 8.40E-04 | 2.058 |
|  | *0.026* | *0.05* | *0.529* | *0.007* | *0.05* | *0.144* |  |  |  |  |

**Scenario 3:** **Field player aged 11 to 18 years (performance-oriented)** (with 11-year old child as worst case)

|  | **Oral** | | | **Dermal *** | | | **Inhalation ^#^** | | | **Total** |
| --- | --- | --- | --- | --- | --- | --- | --- | --- | --- | --- |
| **11-year old child** | **Exposure (µg/kg bw/d)** | **TDI^$^ (µg/kg bw/d)** | **RCR** | **Exposure (µg/kg bw/d)** | **TDI (µg/kg bw/d)** | **RCR** | **Exposure (µg/m^3^)** | **air limit (µg/m^3^)** | **RCR** | **RCR** |
| Cadmium | 0 | 0.36 | 0 | 0.001 | 0.36 | 0.004 | 2.52E-05 | 0.005 | 5.04E-03 | 9.13E-03 |
| Cobalt | 0.002 | 1.4 | 0.002 | 0.035 | 1.4 | 0.025 | 1.20E-03 | 0.5 | 2.40E-03 | 0.029 |
| Lead | 0.010 | 0.05 | 0.201 | 0.005 | 0.05 | 0.103 | 4.20E-04 | 0.5 | 8.40E-04 | 0.305 |
|  | *0.006* | *0.05* | *0.120* | *0.002* | *0.05* | *0.043* |  |  |  |  |

Table S6 – continued

**Scenario 4: Field player aged 18 to 35 years (performance-oriented)**

|  | **Oral** | | | **Dermal *** | | | **Inhalation ^#^** | | | **Total** |
| --- | --- | --- | --- | --- | --- | --- | --- | --- | --- | --- |
|  | **Exposure (µg/kg bw/d)** | **TDI^$^ (µg/kg bw/d)** | **RCR** | **Exposure (µg/kg bw/d)** | **TDI (µg/kg bw/d)** | **RCR** | **Exposure (µg/m^3^)** | **air limit (µg/m^3^)** | **RCR** | **RCR** |
| Cadmium | 0 | 0.36 | 0 | 0.002 | 0.36 | 0.005 | 2.52E-05 | 0.005 | 5.04E-03 | 9.88E-03 |
| Cobalt | 0.001 | 1.4 | 0.001 | 0.042 | 1.4 | 0.030 | 1.20E-03 | 0.5 | 2.40E-03 | 0.033 |
| Lead | 0.007 | 0.05 | 0.131 | 0.006 | 0.05 | 0.122 | 4.20E-04 | 0.5 | 8.40E-04 | 0.254 |
|  | *0.004* | *0.05* | *0.078* | *0.003* | *0.05* | *0.051* |  |  |  |  |

* In the absence of a dermal toxicological reference value, the oral TDI was taken.

# In the absence of content data from own research, the Dutch Milieukeur content values were taken for cadmium and lead (68). Since the Milieukeur data offered no content values for cobalt, a fictitious level of 100 mg/kg was chosen.

$ The toxicological reference value for cadmium is a TWI of 2.5 µg/kg bw/wk; this has been converted into a TDI of 0.36 µg/kg bw/d. For lead, the toxicological reference value is not a TDI, but a maximum exposure level for a non-threshold effect; since RCRs are not applicable for non-threshold substances, the values present the level by which the maximum exposure level are exceeded, not true RCRs.

(RCR = Risk Characterisation Ratio; TDI = Tolerable Daily Intake; TWI = Tolerable Weekly Intake)

Table S7. Results of the risk assessment for bisphenol A (BPA) for exposure scenarios 1, 2, 3 and 4 (A: daily exposure; B: year average exposure) and for scenario 5 (B: ‘lifelong’ exposure goalkeeper); based on maximum pitch values

**A. Daily exposure**

|  |  | **Oral** | | | **Dermal** | | | **Inhalation** | | | **Total** |
| --- | --- | --- | --- | --- | --- | --- | --- | --- | --- | --- | --- |
| **Scenario** * | **max level rubber granulate (mg/kg)** | **Exposure (µg/kg bw/d)** | **DNEL (µg/kg bw/d)** | **RCR** | **Exposure (µg/kg bw/d)** | **DNEL (µg/kg bw/d)** | **RCR** | **Exposure (µg/m^3^)** | **DNEL (µg/m^3^)** | **RCR** | **RCR** |
| **1 (4-yr old)** | 2.5 | 0.032 | 4 | 7.96E-03 | 0.016 | 0.1 | 0.16 | 0.00003 | 200 | 1.50E-07 | 0.17 |
| **2 (7-yr old)** | 2.5 | 0.021 | 4 | 5.14E-03 | 0.103 | 0.1 | 1.03 | 0.00003 | 200 | 1.50E-07 | 1.03 |
| **3 (11-yr old)** | 2.5 | 0.003 | 4 | 6.98E-04 | 0.018 | 0.1 | 0.18 | 0.00003 | 200 | 1.50E-07 | 0.18 |
| **4 (adult)** | 2.5 | 0.002 | 4 | 4.54E-04 | 0.022 | 0.1 | 0.22 | 0.00003 | 200 | 1.50E-07 | 0.22 |

**B. Year average exposure / ‘Lifelong’ exposure**

|  |  | **Oral** | | | **Dermal** | | | **Inhalation** | | | **Total** |
| --- | --- | --- | --- | --- | --- | --- | --- | --- | --- | --- | --- |
| **Scenario** * | **max level**  **rubber granulate (mg/kg)** | **Exposure (µg/kg bw/d)** | **DNEL (µg/kg bw/d)** | **RCR** | **Exposure (µg/kg bw/d)** | **DNEL (µg/kg bw/d)** | **RCR** | **Exposure (µg/m^3^)** | **DNEL (µg/m^3^)** | **RCR** | **RCR** |
| **1 (4-yr old)** | 2.5 | 0.005 | 4 | 1.33E-03 | 0.003 | 0.1 | 0.03 | 0.00003 | 200 | 1.50E-07 | 0.03 |
| **2 (7-yr old)** | 2.5 | 0.007 | 4 | 1.84E-03 | 0.026 | 0.1 | 0.26 | 0.00003 | 200 | 1.50E-07 | 0.26 |
| **3 (11-yr old)** | 2.5 | 0.002 | 4 | 4.15E-04 | 0.008 | 0.1 | 0.08 | 0.00003 | 200 | 1.50E-07 | 0.08 |
| **4 (adult)** | 2.5 | 0.001 | 4 | 2.70E-04 | 0.009 | 0.1 | 0.09 | 0.00003 | 200 | 1.50E-07 | 0.09 |
| **5 (lifelong)** | 2.5 | 0.003 | 4 | 6.99E-04 | 0.009 | 0.1 | 0.09 | 0.00003 | 200 | 1.50E-07 | 0.09 |

* Scenario 1: Field player aged 4 to 11 years (recreational) (with 4-year old child as worst case)

Scenario 2: Goalkeeper from 7 years of age (with 7-year old child as worst case)

Scenario 3: Field player aged 11 to 18 years (performance-oriented) (with 11-year old child as worst case)

Scenario 4: Field player aged 18 to 35 years (performance-oriented)

Scenario 5: ‘Lifelong’ exposure for someone who starts out playing football from age 4-7 and then is a goalkeeper from age 7-50.

(DNEL = Derived No-Effect level; RCR = Risk Characterisation Ratio)
